# Hyponatraemia after Fracture Is Often Caused by Hypovolaemia, Not SIADH

**DOI:** 10.1155/2015/959213

**Published:** 2015-02-24

**Authors:** K. Cumming, R. L. Soiza

**Affiliations:** ^1^School of Medicine & Dentistry, University of Aberdeen, Aberdeen, UK; ^2^Department of Geriatric Medicine, Aberdeen Royal Infirmary, NHS Grampian, Aberdeen, UK

We read with interest the study by Cervellin et al., the largest study of admission hyponatraemia in intracapsular femoral neck fractures to date [1]. This work adds to the substantial and growing body of evidence detailing associations between hyponatraemia and fractures. We agree that hyponatraemia is an important and underappreciated problem in femoral fractures and other fragility fractures but would like to highlight some important issues. Cervellin et al. cite that syndrome of inappropriate antidiuretic hormone (SIADH) causes around 50% of cases of hyponatraemia, with the remaining causes being related to chronic conditions or medication. However, few quality studies have accurately investigated underlying causes of hyponatraemia in people with fractures. We recently conducted a prospective observational study of hyponatraemia in elderly people with fragility fractures (EPFF) and, to our knowledge, have reported the most accurate and in-depth account of aetiology of hyponatraemia in EPFF to date [2]. We note that the group cite our work. However, they state that we found no difference in aetiology of hyponatraemia between fracture and nonfracture patients when we only studied patients with fractures. Our expert panel paid particular attention to volemic status and found that the majority of cases of hyponatraemia were due to hypovolaemic causes rather than SIADH. Cases of hyponatraemia were predominantly multifactorial in aetiology; the commonest causative factors were bendroflumethiazide therapy, dehydration (inadequate fluid replacement), and proton pump inhibitors. SIADH was implicated in only 27% of cases. Our finding that causes of hyponatraemia were predominantly related to hypovolaemia rather than SIADH has important clinical implications because treatment requirements are exact opposites (rehydration or fluid restriction), and inappropriate treatment can have serious consequences, particularly for frail older patients with fractures. Establishing aetiology of hyponatraemia is notoriously challenging and requires accurate assessment of volemic status [3]. Cervellin et al. acknowledge the well known difficulties in clinical management of hyponatraemia and mention the potential use of vaptans. However, we would highlight that these are not appropriate in cases of hypovolaemic hyponatraemia, nor are they licensed in all countries. Furthermore, in normonatremic patients with, or at risk of, fractures, clinicians should take particular care in maintaining appropriate fluid and electrolyte balance and avoid unnecessary use of medications associated with hyponatraemia in order to prevent development of hyponatraemia and its sequelae of poor clinical outcomes. In our study, admission prevalence of hyponatraemia was 13.4% and a further 12.6% developed hyponatraemia during hospital stay. Differences in admission prevalence may be attributable to variation in study sample criterion or characteristics or may be due to Cervellin et al.'s significantly larger sample size. The high incidence of hyponatraemia developing in hospital in our study is important to mention as reportedly those who develop or experience worsening hyponatraemia during hospital stay fare worse than those who are hyponatremic on admission [4, 5]. In addition to further falls and fractures, hyponatraemia is also associated with longer hospital stay, mortality, and institutionalization [4]. This highlights the importance of preventing development of hyponatraemia, particularly in patients with, or at risk of, fractures.
